# Analysis of Postsurgical Health-Related Quality of Life and Quality of Voice of Patients With Laryngeal Carcinoma

**DOI:** 10.1097/MD.0000000000002363

**Published:** 2016-01-08

**Authors:** Jie Luo, Jieli Wu, Kexing Lv, Kaichun Li, Jianhui Wu, Yihui Wen, Xiaoling Li, Haocheng Tang, Aiyun Jiang, Zhangfeng Wang, Weiping Wen, Wenbin Lei

**Affiliations:** From the Otorhinolaryngology Hospital, Otorhinolaryngology Institute, The First Affiliated Hospital, Sun Yat-Sen University, Guangzhou, Guangdong, China (JL, JL W(Jieli Wu), KX L(Kexing Lv), YW, HT, AJ, ZW, WW, WL); Tianyou Hospital Affiliated to Tongji University, Shanghai, China (JL, KC L(Kaichun Li)); Otorhinolaryngology Institute, Meizhou People's Hospital, Meizhou, Guangdong, China (JW); and The Fourth People's Hospital of Shenzhen (Affiliated Futian Hospital of Guangdong Medical College), Shenzhen, China (XL).

## Abstract

This study aims to analyze the postsurgical health-related quality of life (HRQOL) and quality of voice (QOV) of patients with laryngeal carcinoma with an expectation of improving the treatment and HRQOL of these patients.

Based on the collection of information of patients with laryngeal carcinoma regarding clinical characteristics (age, TNM stage, with or without laryngeal preservation and/or neck dissection, with or without postoperative irradiation and/or chemotherapy, etc.), QOV using Voice Handicap Index (VIH) scale and HRQOL using EORTC QLQ-C30 and EORTCQLQ-H&N35 scales, the differences of postsurgical HRQOL related to their clinical characteristics were analyzed using univariate nonparametric tests, the main factors impacting the postsurgical HRQOL were analyzed using regression analyses (generalized linear models) and the correlation between QOV and HRQOL analyzed using spearman correlation analysis.

A total of 92 patients were enrolled in this study, on whom the use of EORTC QLQ-C30, EORTC QLQ-H&N35 and VHI scales revealed that: the differences of HRQOL were significant among patients with different ages, TNM stages, and treatment modalities; the main factors impacting the postsurgical HRQOL were pain, speech disorder, and dry mouth; and QOV was significantly correlated with HRQOL.

For the patients with laryngeal carcinoma included in our study, the quality of life after open surgeries were impacted by many factors predominated by pain, speech disorder, and dry mouth. It is suggested that doctors in China do more efforts on the patients’ postoperative pain and xerostomia management and speech rehabilitation with the hope of improving the patients’ quality of life.

## Abstract 2

Supplemental Digital Content is available in the text

## INTRODUCTION

As a commonly seen head–neck malignancy, laryngeal carcinoma in south China has an incidence that is just secondary to that of nasopharyngeal carcinoma and has shown a trend of increase during recent year. At present, the mainstream of treatment of laryngeal carcinoma in China is surgery, complemented with radio- or chemotherapy. Being a promising curative method, surgery can also cause pains and perplexes to patients, such as speech disorder, aspiration, and even dyspnea with respect to the unique anatomic site and physiological functions of the larynx. On the other hand, the use of adjuvant radio- and/or chemo-therapy can cause adverse effects such as xerostomia, inflammation, and ulceration of the mucosa and skin, which can lead to further depression of the patients’ postsurgical social functions and overall health statuses.^1,2^ Thus, a thorough evaluation of the patients’ postsurgical quality of life (QOL) is hopefully helpful for the doctors to optimize the treatment regimens and improve the therapeutic outcomes with fewer pains and eventually better QOL of these patients.

QOL is defined as a person's the self-cognition as an individual existing in a certain system of culture and value, his comprehensive evaluation of his own physical health, psychologic status, social connections, and belief.^3^ The evaluation of QOL requires an instrument that is comprehensive and multi-dimensional, standardized and easily understandable, and workable.^4–6^ The health-related quality of life (HRQOL) scaling system established by the European Organization for Cancer Research and Treatment (EORTC) is 1 of the tools that meet all the criteria mentioned above and has been used worldwide in the studies of HRQOL of patients with cancer. Our present study employs three scales: EORTC QLQ-C30, EORTC QLQ-H&N35,^7^ and Voice Handicap Index (VHI),^8^ to evaluate patients’ postsurgical HRQOL and quality of voice (QOV) related to their clinical characteristics; We also calculated the correlation between HRQOL and QOV. The aim is to remind clinical doctors that with the survival rate having been so much focused on, the improvement of QOL of patients with laryngeal carcinoma should also be achieved by optimizing the treatment of this disease.^9^

## METHODS

### Patients

The personal information (name, age, gender, address, and contact method) and clinical information (surgical modality as total laryngectomy or partial laryngectomy, larynx preserved or not, with or without postoperative irradiation, with or without chemotherapy, UICC stage I–II/III–IV) of the patients meeting the inclusion criteria described later were collected. All the patients underwent surgery in our hospital except 32 patients who underwent total laryngectomy in other hospitals in Guangdong Province and who are also members of The New Voice Club of Guangzhou, a nonprofit organization of laryngectomees.

Inclusion criteria: those who had had an open surgery at least 12 months before and had been pathologically diagnosed with squamous cell carcinoma of the larynx; those who volunteered for follow-up and were able to understand the questionnaires; those who were in good health during the follow-up, without mental disorders, cognitive disorders, or other severe systemic diseases.

Exclusion criteria: those who had comorbid severe systemic diseases such as other malignancies; those whose personal information was not completed or who were incapable of completing the questionnaires (except for items 29 and 30 of EORTC QLQ-H&N35 which are related to sexuality).

This study was approved by the Ethics Committee of the First-Affiliated Hospital of Sun Yat-Sen University.

### Scales

The HRQOL of patients with laryngeal carcinoma was estimated with EORTC QLQ-C30 and EORTC QLQ-H&N35. EORTC QLQ-C30 is the core scale of HRQOL of patients with cancer including 5 functional domains (physical, role, emotional, cognitive, and social) composed of 3 symptom scales (fatigue, nausea and vomitus, and pain), 1 domain related to overall status of survival (overall health and overall QOL), and 6 single-symptom items (insomnia, shortness of breath, inappetence, constipation, diarrhea, and financial difficulty). The 2 scales in the overall survival status are scored from 1 (very poor) to 7 (excellent) and all the items in the other domains are scored from 1 (not at all) to 4 (very much). EORTC QLQ-H&N35 for head–neck cancers is composed of 35 items reflecting head–neck symptoms which are divided into 7 domains (including pain, odynophagia, cognitive disorder, speech disorder, eating difficulty, human communication disorder, and impact on sexuality) and 11 single-symptomatic items. The first 30 items are scaled from 1 (not at all) to 4 (very much) and the last 5 are only scaled as 1 for “yes” and 0 for “no.” The primary scores of both the scales should be converted according to the EORTC QLQ-C30 grading manual (Version 3) to centesimal system,^10^ where higher functional scores and/or lower symptomatic scores are related to better HRQOL. Both the 2 scales were validly downloaded from the official website of EORTC (http://groups.eortc.be/qol/) and their Chinese versions had been tested for both credibility and validity in Chinese patients with cancers, promising its use for evaluation of HRQOL of Chinese patients.^11,12^

VHI is accepted as the “gold standard” for the self-evaluation of QOV of patients with voice disorders.^13^ A total of 30 items in VHI scale are divided into 3 domains (VHI functional, VHI physical, and VHI emotional), each of which contains 10 items scaled from 0 (never) to 4 (always). Thus a summation of all the scores of the 4 domains (0–40 for each) as the total score ranged from 0 to 120 can be used as a self-evaluation of the patient's QOV, where the higher the score is, the more unsatisfied the patient is with his/her own voice.

### Statistical Methods

All the statistical analyses were processed with SPSS 18.0 software. The distribution of scales’ scores was not normal (Kolmogorov–Smirnov test *P* < 0.05). Mann–Whitney *U* test was used to analyze the differences of postsurgical HRQOL and QOV among patients with different clinical characteristics. The correlation of global health/QOL in QLQ-C30 with the 3 VHI domains/total scores was analyzed using Spearman correlation analysis. The 13 symptomatic scores (including 6 domains and 11 single items) of QLQ-H&N35 as the independent variables and the global health/QOL of QLQ-C30 as the dependent variable were all put into a generalized linear models (GLM) equation by a stepwise way, where *P* < 0.05 was considered statistically significant.

## RESULTS

There were initially 132 patients who met our inclusion criteria and were followed-up in our hospital from August 2012 to August 2013. The object and the flowchart of our study was introduced to the patients by a specially trained clinical doctor who later on signed the informed consent with them and conducted them by one-to-one to complete the 3 scales. With those who met the exclusion criteria excluded, a total of 92 patients were eventually studied, with balanced and comparable data except for gender (Table 1).

**TABLE 1:**
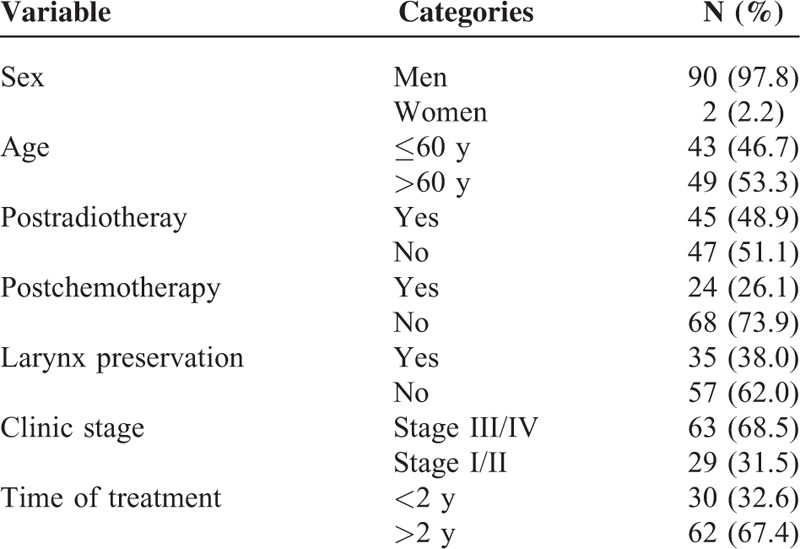
Characteristics of Patients

### Differences of HRQOL Among Patients With Different Clinical Characteristics

It was revealed by statistics that in 2 types patients (ie, patients above 60 years [*P* = 0.030] and patients with postoperative adjuvant chemotherapy [*P* = 0.003]) claimed worsened postsurgical HRQOL. (Of the whole sample, the median age is 60 years, the mean age is 59.88 years with a standard deviation of 8.78 years.) Compared with the younger patients (ages <60), the elder patients (ages ≥60) were poorer in multiple symptoms such as that they needed more analgesics and nourishment but better in social functions and financial statuses. The clinical stage played a role in largely aspects of the patients’ HRQOL. Late-staged patients reported 4 aspects of QLQ-C30 and 7 of QLQ-H&N35 poorer than early-staged patients. The laryngectomees reported significantly different HRQOL from their larynx-preserved counterparts in that they commonly complained dysphagia (*P* = 0.025) and sense problems (*P* = 0.000), limitations in social dieting (*P* = 0.003) and social contact (*P* = 0.000). Patients with postoperative irradiation had poorer HRQOL than patients without postoperative irradiation in 2 aspects (social function and fatigue) of QLQ-C30 and 6 (pain, swallowing, sensory disturbances, social dieting, social contact, and xerostomia) of QLQ-HN35. Similarly, postoperative chemotherapy was also related to poorer HRQOL, mainly regarding 3 symptomatic domains (pain, vomitus, and diarrhea) of QLQ-C30 and 7 (dysphagia, sensory and speech disorders, social dieting and contact limitation, dental problems, and xerostomia) of QLQ-H&N35. There were also some differences in symptoms related to other factors, such as neck dissection and follow-up time (Tables 2 and 3).

**TABLE 2:**
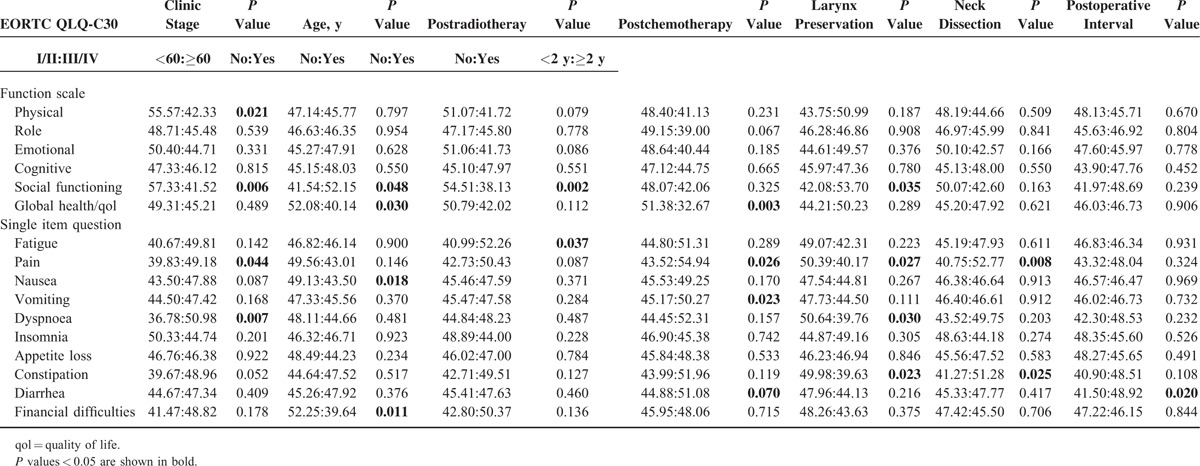
Comparison of Domains in EORTC QLQ-C30 Affected by Various Clinic Conditions

**TABLE 3:**
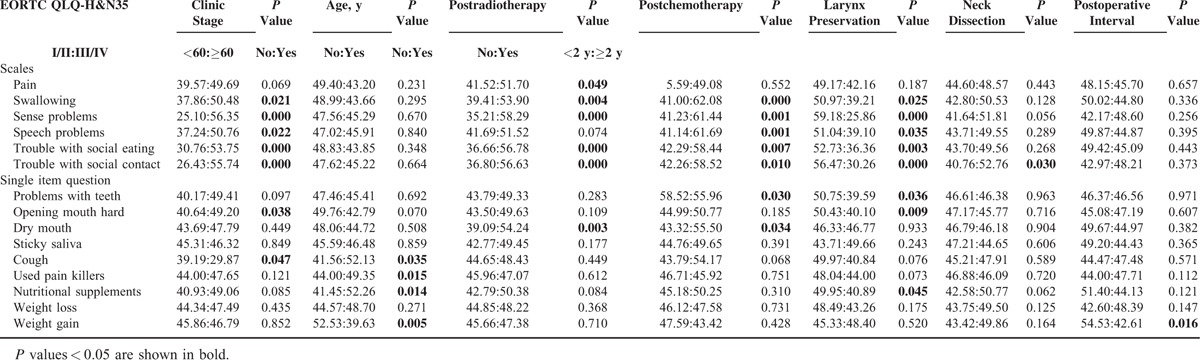
Comparison of Domains in EORTC QLQ-H&N35 Affected by Various Clinic Patients’ Characteristics

### QOV of Patients With Different Clinical Characteristics

The scores in all the domains of VHI scale were compared in order to explore the differences in postsurgical QOV among laryngeal carcinoma patients with different clinical characteristics and their impacting factors. As is seen in Table 4, clinical stage, postoperative irradiation, postoperative chemotherapy, and preservation of larynx are all significantly correlated to differences in QOV which were, instead, not correlated to age, neck dissection, or postoperative follow-up time (Table 4).

**TABLE 4:**
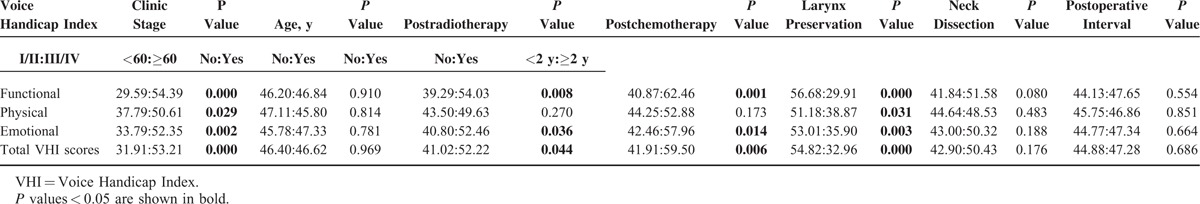
Comparison of Domains in VHI Affected by Various Clinic Patients’ Characteristics

### Leading Factors Impacting Global Health/QOL

The 13 symptomatic scores (including 6 domains and 7 single items) of QLQ-H&N35 as the independent variables and the global health/QOL of QLQ-C30 as the dependent variable were all put into a GLM equation, in order to explore the leading factors impacting the postsurgical HRQOL of patients with laryngeal carcinoma. Before it, we had assessed the correlation between global-QOL and domains in QLQ-H&N35 through Spearman correlation method, which usually pioneers or provides some reference for GLM analysis (Supplemental Table). Finally, the results revealed that pain, speech disorder, dry mouth, and weight gain were significantly related to global health/QOL (*P* < 0.05; Table 5).

**TABLE 5:**
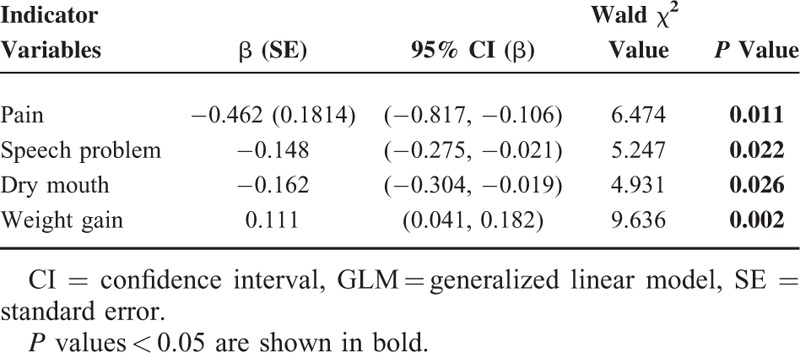
Multivariable Models (GLM) Evaluating the Relationship Between EORTC QLQ-H&N35 Indicator Variables and Global Health/QOL

### Correlation Between Global Health/QOL and QOV

**FIGURE 1:**
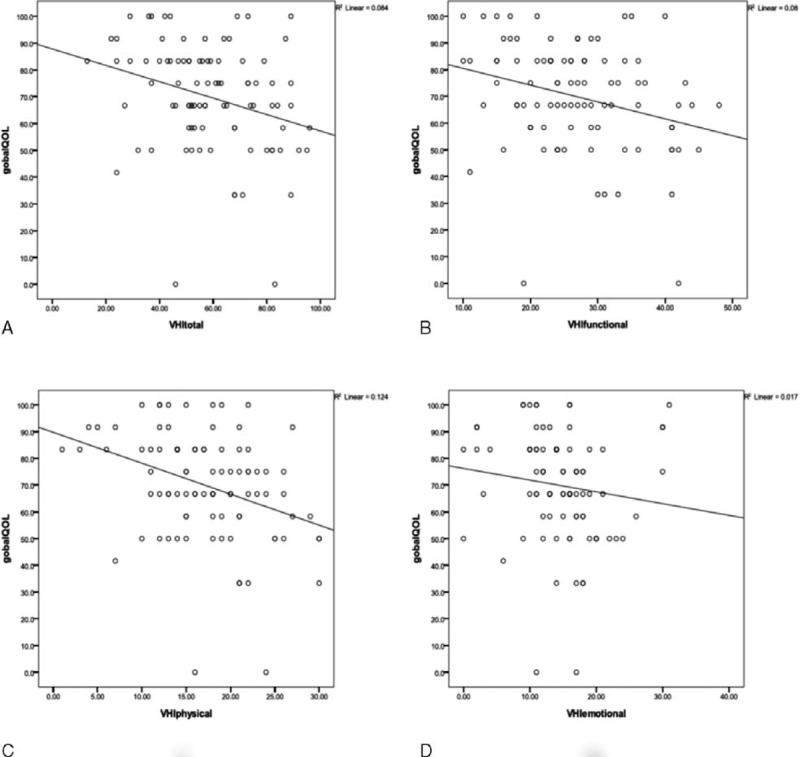
Scatter plot analysis of the relationship between VHI sub-domains (A, Total; B, Functional; C, Physical; D, Emotional) and Global HRQOL. HRQOL = health-related quality of life, VHI = Voice Handicap Index.

It was revealed by Spearman correlation analysis that functional, physical, and total VHI scores were all negatively correlated with global health/QOL of QLQ-C30 (*P* < 0.01), while emotional VHI score was not (*P* = 0.214). This demonstrated the close correlation between QOV and HRQOL (Table 6; Figure 1).

**TABLE 6:**
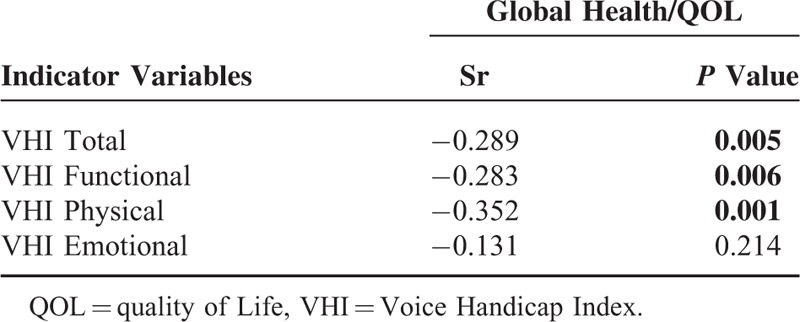
Spearman Correlations Between Global Health/QOL and VHI Domains

## DISCUSSION

### Employment and Characteristics of Clinical Data and QOL Scales in Our Study

The clinical data of 92 patients out 132 followed-up patients were enrolled in our study, with a follow-up rate of 70%, which is higher than some other similar studies.^14^ Our study is also characterized by a relatively high rate of laryngectomees (62%) partly because of the patients from the New Voice Club of Guangzhou, which is a nonprofit organization aiming to help laryngectomees regain speech and get adapted to the physical and psychological changes related to laryngeal carcinoma. In that case, we could study more about the QOV and HRQOL of laryngectomees. The researchers were specially trained for one-to-one follow-up of patients so that the consistency of follow-up outcome was effectively improved. Being the HRQOL scale exclusively for head–neck cancer patients, EORTC QLQ-H&N35 (Version in Chinese)^12^ was used in combination with and as a supplement of EORTC QLQ-C30, as is often seen in other studies.^7^ These 2 scales which were introduced into China and translated into Chinese during recent years crown all the aspects regarding the HRQOL of patients with cancers and are easy to understand and fill in, showing good validity and credibility.^15,16^ In spite of that, these 2 scales are not sufficient for the evaluation of QOV which, however, can be well evaluated using VHI,^17^ implying the emphasis on QOV of our study. The correlation between QOV and HRQOL of patients with laryngeal carcinoma was not so much studied in the past and the results of the previous studies were dramatically different from one another. Lundstrom et al^17^ concluded that total VHI and emotional VHI were both significantly correlated to global health/QOL, but Stewart et al^18^ concluded not. The present change of medical mood from biomedical mood to biopsychosocial mood in China requires more emphasis on the QOL of patients with laryngeal carcinoma, which, however, is somehow neglected by Chinese doctors and not so adequately reported. So, it is hoped that our study can somehow fill the gap in this field.

### Differences in HRQOL Among Patients With Different Clinical Characteristics

The impact of age on the HRQOL of patients with cancer has been confirmed in many previous studies.^12,19^ Our analysis using EORTC QLQ-C30 and QLQ-H&N35 can draw a similar conclusion that elder patients are more vulnerable to symptoms caused by cancer, as was seen in multiple domains or aspects, leading to a significantly poorer global health/QOL than that of young patients. Nevertheless, young patients are more concerned about the impacts of cancer on their social functions and financial statuses probably because of their limited economic strengths but bigger social/familial responsibilities so that it is more difficult for them than for the elder patients to get adapted to the social and economic pressures caused by cancer.

Patients with laryngeal carcinoma who receive postoperative chemotherapy will stand many kinds of discomforts including xerostomia, pain, vomitus, diarrhea, etc.^20^ However, many reports^21,22^ concluded that postoperative chemotherapy had no significant impact on the patients’ global health status. In our study, the patients with postoperative chemotherapy had poorer global health/QOL, as well as the HRQOL in 2 aspects (social functions and fatigue) of QLQ-C30 and 6 (pain, swallowing, sensory disturbances, social dieting, social contact, and xerostomia) of QLQ-HN35. This could have been related to the oriental race that cannot bear chemotherapeutic regimens with such big dosages which, at present, have been established mainly according to foreign clinical data. A prospective study completed in China has recommended decreased drug dosages in TPF neoadjuvant chemotherapy against late-staged head–neck cancers (Taxotere 60 mg/m^2^ d1, DDP 60 mg/m^2^ d1, 5-FU 600 mg/m^2^ d1–5 repeated in 3-week cycles), which seems safer, more efficient, and more appropriate for easterners.^23^ It is suggested that besides efficacy, the impact of postoperative chemotherapy on HRQOL be also taken into consideration with dosages appropriate for easterners so as to improve HRQOL by reducing adverse effects.

### Global Health/QOL and Its Leading Impacting Factors

As a unique domain of EORTC QLQ-C30, global health/QOL is a comprehensive judgment made by the patient him/herself on his/her own physical status and state of life which reflects the patient's satisfaction and general feeling about his or her post-therapeutic life. However, global health/QOL was previously seen only as simply a scoring other than a key to explore the underlying functional deficiency and symptomatic disturbances.

Our study combined the 2 different scales using GLM analysis so that both systemic and regional statuses were estimated, drawing a conclusion that could better reflect the patients’ actual clinical states. Our study demonstrated that pain, dry mouth, and speech disorder were the main factors impacting the postsurgical HRQOL of patients with laryngeal carcinoma. Nevertheless, Lundstrom et al^24^ concluded that global health/QOL was related to xerostomia, swallowing disorders, human communication disorders and so on, not to dry mouth, and speech disorders. This is probably because that there was a high proportion of laryngectomees in our study and that we Chinese have, from that of westerners, different economic, cultural and familial conceptions, physical conditions, and treatment privileges, which can all influence our choice of treatment modalities. People in western countries have better medical insurance, so that most of the patients with laryngeal carcinoma were treated with larynx preserving methods such as irradiation, chemoradiotherapy, and partial laryngectomy with postoperative irradiation. When it comes to our patients, most of them were treated with surgery which is relatively cheap and less toxic, mainly aiming at longer survival. Laryngectomees were in a high proportion with low rates of postoperative phonatory button installation and esophageal phonation, so that they had to use electrolarynges for communication, leading to poor QOV dramatically influencing HRQOL.

It was demonstrated in our study that postsurgical QOV was significantly negatively correlated with global health-related QOL (Figure 1), implying that QOV was an important factor impacting HRQOL. So, improvement of QOV must be thoroughly considered when making a treatment planning for a patient with late-staged laryngeal carcinoma. For these patients especially those who strongly demand preservation of voice, organ preservation therapies should be given with the permission of global health and oncological statuses. These therapies include partial laryngectomy followed by postoperative irradiation, preoperative chemotherapy or irradiation followed by preservative laryngectomy or chemoradiotherapy according to the response of tumor, or chemoradiotherapy directly used as a curative method. For those whose larynges must be sacrificed, an attempt of simultaneous voice restoration must be tried with methods such as Blom-Singer tube to improve postsurgical HRQOL.

According to the GLM analysis in our study, the other important factor that significantly impacted the postsurgical HRQOL of patients with laryngeal carcinoma was pain. Nowadays, in most of the hospitals in China, open surgeries including laryngectomies and neck dissections are still the mainstream therapy against laryngeal carcinoma, with adjuvant irradiation and/or chemotherapy for late-staged diseases. Under this situation, postsurgical pain can be caused by multiple factors, obviously depressing the HRQOL of patients. In western countries, however, irradiation or chemoradiotherapy are used against laryngeal carcinoma as the main treatments, causing relatively mild adverse effects only in a short term. This could have explained the difference of our outcome from that of similar studies in other countries. Pain can cause not only worsening of HRQOL of the patients^25,26^ but also an adverse psychological suggestion with a negative impact on the patients’ living attitudes and statuses. Therefore, on one hand, surprising outcomes may be achieved by providing the patients’ with pain with psychological counseling as well as analgesics; on the other hand, all efforts must be done in the treatment planning for late-staged laryngeal carcinoma to preserve as many normal tissues and organs as possible, in addition to radical therapy, so as to reduce, to the maximum extent, surgical trauma. Intraoperative techniques such as detection of sentinel lymph nodes are advocated to avoid unnecessary neck dissection. The dosages and courses of adjuvant radio- and chemotherapy should be well profiled in order not to add too much to the surgical trauma causing pain.

Weight gain was demonstrated in our study as a positive impacting factor for HRQOL. This may be because that weight gain has been traditionally considered by Chinese as an improvement of global health and some of the elder patients even consider it the sign of regaining of health. As unique as it is, the positive impact of weight gain on the HRQOL of our patients was significant.

### Limitations and Future Views of Our Study

The main limitation of our study is that only 30% of the patients replied in the domain of sexuality in their questionnaires, making it insufficient for further statistical analysis. Most of the patients were reluctant to this issue when asked about their status of sex, which was typical in the Chinese culture. Despite that sexuality is much more openly accepted as part of HRQOL by the public in China, it is still, to many Chinese, a topic not so easily sharable with others, not even doctors or other professionals. Thus, the meaning of normal sexuality to the improvement of HRQOL is often neglected, either actively or passively. Above all, we had to give up the exploration in this domain. To better this situation, doctors must try to get trusted by and further know about their patients so that appropriate guidance can be given to help improve the patients’ HRQOL.

Other limitations also exist in our study. First of all, this is a retrospective cross-sectional study in which the lack of information of the patients’ preoperative health statuses made difficult a preoperative–postoperative comparison. Therefore, the statistical results should not be contributed only to the treatment modalities. On the other hand, this is a short-term follow-up study that cannot enable survival analysis. Third, the high expenses and relapse rates of irradiation and chemoradiotherapy made them not yet so widely accepted in China so that these patients who are in a relatively small number were not included in our study.

QOL is not only a series of numbers but an important concept that must be kept in every doctor's mind.^27^ For patients with laryngeal carcinoma, whose function-related QOL can probably be further improved by phonation training and psychological counseling.^28^ Some surgeons are trying work out more skillful surgeries aiming at better QOV, from which may further improve the patients’ postsurgical global health/QOL.

## CONCLUSION

For the patients with laryngeal carcinoma included in our study, the QOL after open surgeries were impacted by many factors predominated by pain, dry mouth, and speech disorder. It is suggested that doctors in China do more efforts on the patients’ postoperative pain management and speech rehabilitation with the hope of improving the patients’ overall quality of life.

## Supplementary Material

Supplemental Digital Content
