# Supplementary material for: Analysis of Postsurgical Health-Related Quality of Life and Quality of Voice of Patients With Laryngeal Carcinoma
Source: Medicine (Baltimore). 2016 Jan 8;95(1):e2363. doi: 10.1097/MD.0000000000002363 (PMC4706258; doi:10.1097/MD.0000000000002363)
Supplement: Supplemental Digital Content [file medi-95-e2363-s001.doc]

Article title: Analysis of Post-surgical Health-related Quality of Life and Quality of Voice of Patients with Laryngeal Carcinoma

First author: Jie Luo

**Supplemental Table. Spearman Correlations between Global health/QOL and EORTC QLQ-H&N35 Indicator Variables**

| **Indicator Variables** | **Global health/QOL** | |
| --- | --- | --- |
|  | ***S_r_*** | ***P*-value** |
| **Pain** | **-0.239** | **0.023** |
| **Swallowing** | **-0.178** | **0.091** |
| **Sense problems** | **-0.220** | **0.036** |
| **Speech problems** | **-0.282** | **0.007** |
| **Trouble with social eating** | **-0.234** | **0.026** |
| **Trouble with social contact** | **-0.232** | **0.027** |
| **Felt ill** | **-0.004** | **0.973** |
| **Problems with teeth** | **-0.221** | **0.035** |
| **Opening mouth hard** | **-0.070** | **0.512** |
| **Dry mouth** | **-0.393** | **＜0.001** |
| **Sticky saliva** | **-0.276** | **0.008** |
| **Cough** | **-0.342** | **0.001** |
| **Used pain killers** | **-0.126** | **0.233** |
| **Nutritional Supplements** | **-0.070** | **0.510** |
| **Used feeding tube** | **0.026** | **0.804** |
| **Weight loss** | **-0.129** | **0.222** |
| **Weight gain** | **-0.198** | **0.060** |
| **QOL= Quality of Life** | | |
